# Influence of nurse and midwife managerial leadership styles on job satisfaction, intention to stay, and services provision in selected hospitals of Rwanda

**DOI:** 10.1186/s12912-020-00428-8

**Published:** 2020-05-06

**Authors:** Anaclet Ngabonzima, Domina Asingizwe, Kyriakos Kouveliotis

**Affiliations:** 1 Training Support Access Model for Maternal Newborn and Child Health (TSAM), Kigali, Rwanda; 2 College of Medicine and Health Sciences, University of Rwanda, Kigali, Rwanda; 3 International Telematic University Uninettuno, Rome, Italy

**Keywords:** Leadership styles; job satisfaction, Intention to stay, Path-goal leadership, Supportive, Directive, Achievement-oriented, Participative

## Abstract

**Background:**

Nurses and midwives are a critical part of the healthcare team and make up the largest section of health professionals. Leadership styles are believed to be an important determinant of job satisfaction and retention making effective leadership within nursing and midwifery crucial to health systems success. In Rwanda, there are gaps in knowledge of managerial leadership styles of nurses and midwives and the influence of these styles on job satisfaction and retention for nurses and midwives who report to them, as well as their influence on the provision of health services. This study describes the managerial leadership styles adopted by nurses/midwives and examines the relationship between managerial leadership styles and job satisfaction, intention to stay, and service provision.

**Methods:**

The Path-Goal Leadership questionnaire was adopted and used to collect data on leadership styles while other questionnaires with high validity and reliability were used to collect data on job satisfaction, intention to stay and service provision. The study involved 162 full-time nurses and midwives practicing in 5 selected hospitals with a minimum of 6 months of experience working with their current direct managers. Regression analysis was used to draw conclusions on relationships between variables.

**Results:**

Nurses and midwives managers were more inclined to the directive leadership style followed by a supportive leadership style, and the participative leadership style. The nurse and midwife’s managerial leadership styles together significantly explained 38, 10 and 23% of the variance in job satisfaction, intention to stay and service provision, respectively.

**Conclusion:**

The findings of this study indicate that managerial leadership styles play a substantial role in enhancing job satisfaction, intention to stay and service provision.

**The implication for nursing and midwifery management:**

There is a need to develop a comprehensive formal professional continuous development course on leadership styles and ensure that all nurses and midwives managers benefit from this course prior to or immediately after being appointed as a manager. Having such a course may even prepare future leaders for their role early in their career. Effective leadership in nursing and midwifery should be enhanced at all levels to improve the job satisfaction of nurses and midwives, address the issue of retention in their respective health facilities and strengthen service provision.

## Background

Job satisfaction and esprit de corps of health care professionals are of concern worldwide [1–7]. Satisfied employees are more likely to perform well and have reduced levels of absenteeism and voluntary turnover [7–9]. Job satisfaction is a strong predictor of overall individual well-being [7, 8, 10] as well as a strong predictor of an employee intention to leave a job [8, 9]. In addition, research suggests that job satisfaction and job performance are positively correlated, and are therefore an issue of substantial importance for both employees and employers [9, 11, 12]. Many studies suggest that employers benefit from lower employee turnover and higher productivity if their employees experience a high level of job satisfaction [8, 9, 11, 12].

When applied to healthcare organizations, job satisfaction not only impact job performance but is also an indicator of efficient service provision and patient satisfaction [9, 11, 13]. Nurses and midwives constitute the largest group of health professionals and are often the frontline health care providers [14]. Job satisfaction is a multidimensional concept and includes not only the employee’s happiness with the leadership behaviour of their supervisors, but also the benefits provided, remuneration, career opportunities, and relationships with their workmates [15, 16].

Once recruited, nurse and midwife managers are responsible for the retention of their frontline nursing and midwifery staff. With limited resources, the right leadership style could potentially play a significant role in the retention of staff and their job satisfaction hence high productivity. Consequently, nursing and midwifery leadership is searching for validated approaches to improve both client and staff outcomes [9, 11–13, 17]. Accordingly, a specific leadership style or a combination of leadership styles is necessary to address the multitude of nursing and midwifery staff and service provision issues [11, 17, 18].

In contrast to other leadership theories that try to identify the dominant leadership styles, the contingency theory of leadership states that effective leaders can use more than one leadership style in response to job satisfaction and motivational needs of their team members [19]. According to this theory, an effective leader should know which style to practice and when [20]. The Path-Goal theory, which also belongs to the contingency model of leadership, identifies four leadership behaviours that increase team members’ satisfaction and intention to stay on the path of goal achievement. These four leadership styles are participative, supportive, directive, and achievement-oriented [19].

**Fig. 1:**
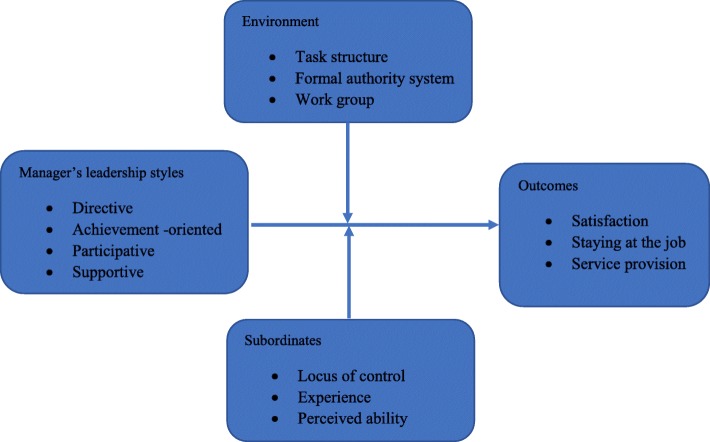
The modified Path-Goal Theory Conceptual Framework (House, 1971). This Figure represents the conceptual framework used in this study as adapted from the Path-Goal Theory

Although there is existing literature on leadership in nursing and midwifery and the factors which affect nurses or midwives’ job satisfaction and intention to stay in their workplaces [21, 22], there are very few of such studies explaining the negative or positive effects of leadership styles on job satisfaction or on intention to stay and health service provision. In Rwanda specifically, no study has focused on managerial leadership styles of nurses and midwives and none have explored how the managerial leadership styles of the latter, affects in one way or the other, job satisfaction, intention to stay and health service provision. This study which used the Path-Goal Leadership theory [23, 24] as an organizing framework (Fig.1), describes the managerial leadership styles used by nurses and midwives, and explores the influence of nurse and midwife’ managerial leadership styles on nursing and midwifery staff job satisfaction, their intention to stay at the hospitals they are currently working for, and health service provision in order to make recommendations that would lead to improved performance of human resource managers in the health sector.

## Methods

### Study area

The study was conducted in five hospitals in the Northern Province of Rwanda, namely Byumba, Kinihira, Ruli, Nemba and Rutongo. These hospitals, located in three districts include public hospitals (Byumba, Rutongo, and Kinihira) and faith-based hospitals (Nemba and Ruli). In addition, one of these hospitals is a provincial hospital (Kinihira), while the other four are district hospitals. While Byumba hospital is an urban hospital, the others are considered rural health facilities based on their location. Given the variety of hospitals in the study, findings may be generalized to other hospitals in Rwanda as most are similar to the hospitals under this study.

### Study design and approach

This study used a descriptive cross-sectional design with a quantitative approach to explore nurses’ and midwives’ perceptions about their managers’ leadership styles and how the latter influences their job satisfaction, intention to stay in their current workplaces and service provision.

### Study population and sample

A multistage-sampling method was used as follows: (1) random sampling of one province to represent others, (2) purposive sampling of three districts to represent the most rural, semi-urban and urban districts, and all hospitals in those three districts were selected, (3) as each hospital had a different number of nurses and midwives, proportional quota sampling was used to get the number of nurses and midwives based on staff population at each hospital, and (4) convenient sampling to recruit nurses and midwives meeting the inclusion criteria and consenting to participate in the study.

During the study period, they were 292 nurses and midwives non-managers in five hospitals in three selected districts (N). Using this total population of nurses and midwives (N) and an alpha level of 0.05, the sample size (n) was calculated using the Taro Yamane simplified sample size scientific formula being cognizant that we had a finite population. The calculated sample size was 169 nurses and midwives. In addition, 10% was added to account for non-response, amounting to a sample size of 185 nurses and midwives.

To ensure that participants knew their managers well enough to provide reliable information on their managerial leadership styles, the study included those with at least six months working experience with their current nurse or midwife manager. Six months was considered a reasonable period during which a nurse or midwife would have enough information related to his/her direct supervisor. Nurses and midwives working in the studied hospitals for non-clinical activities and those working as students, interns or volunteers were excluded.

### Data collection instrument

To suit the objectives and context of this study, existing standard tools [11] were adjusted slightly to accommodate relevant changes. The questionnaire was divided into five sections as follows: Section A was used to collect socio-demographic data; section B held the contents of the Path-Goal Leadership tool which had twenty items used to measure nurse or midwife managerial leadership’s styles through a five-point Likert’s scale. With scores ranging from five to twenty-five, each managerial leadership style was measured using five items. Scores below 12 suggested non-use of a specific managerial leadership style; scores between 12 and 20 indicated a moderate use of a specific leadership style; while scores above 20 suggested a high use of a specific managerial leadership style. Section C served to assess the job satisfaction of nurses or midwives and it had seven items. Higher scores suggested a higher level of job satisfaction. Section D contained four items that elicit nurses or midwives’ intention to stay in their current hospitals with higher scores reflecting higher intentions to stay. Finally, section E included questions used to collect data on perceptions of service provision by nurses and midwives to help measure the influence of other variables on service provision. Higher scores reflected a higher level of service provision.

To ensure the validity of the instrument, a pre-test of the data collection tools was conducted with 10 nurses and midwives from a different health facility (hospital) for identification and modification of areas of misunderstanding in the research tools.

### Data collection

Following the approval of the research protocol by the University of Rwanda- College of Medicine and Health Sciences Institutional Review Board (CMHS-IRB), a copy of the research questionnaire and ethics approval was sent to each of the hospitals for their perusal and to gain permission to conduct the study in their respective hospitals. Five research assistants (one in each hospital) were trained in data collection and when permission was formally granted by a hospital, the PI or the research assistant went to the hospital to personally administer the questionnaire. In each facility, the Director of Nursing (DN) was contacted and helped the investigator identify prospective participants. Nurses/Midwives who met the inclusion criteria and agreed to participate in the study received detailed information about it and were requested to voluntarily participate by signing a consent form. The questionnaires were then distributed and returned to the PI as soon as they were completed. For the staff to be considered a nurse or midwife manager, s/he had to be the direct supervisor who serves as someone to report to as per the principle of management of Henri Fayol related to the Unit of Command [25].

### Statistical analysis

Data were analyzed using the Statistical Package for Social Sciences (SPSS) version 21. Descriptive statistics were used to summarize the data. To determine relationships between variables, multiple regression analyses, and the Pearson Product Moment Correlation were used to establish the relationships between various managerial leadership styles and job satisfaction, as well as staff intention to stay in the current hospital and perceptions on service provision.

## Results

### Sociodemographic characteristics of respondents

Among 185 participants who were recruited in this study, 162 completed and returned the questionnaires, representing a response rate of 87.5%.

With a modal age of 32 years, the mean age of the participants was 35.25 (SD = 6.08) years. Of all 162 respondents, 54.1% of the were aged between 30 and 40 years, while only less than 3% were more than 50 years old. The majority of participants (80.3%) were practicing in district hospitals with 51.9% from faith-based hospitals and 28.4% from the public district hospitals. Most of the participants (61.2%) were female. Nurses A1 constituted the majority of participants (59.3%) and only 6.8% were nurses A2. The details of the demographic characteristics of the participants are presented in Table 1.

**Table 1:** Sociodemographic characteristics of respondents

Variables	Categories	Frequency	Percent
Hospital type	District public	46	28.4
	District faith-based	84	51.9
	Provincial	32	19.8
	Total	162	100.0
Age category of participant	20–30	25	18.5
	30–40	73	54.1
	40–50	33	24.4
	50 and above	4	3.0
	Total	135	100.0
Gender	Male	62	38.8
	Female	98	61.2
	Total	160	100.0
Qualification of the participants	Nurse A2	11	6.8
	Nurse A1	96	59.3
	Nurse A0	24	14.8
	Midwife A1	29	17.9
	Others	2	1.2
	Total	162	100.0
The unit where the participant works	Out Patient Department	22	13.6
	Maternity	34	21.0
	Theatre	9	5.6
	Pediatric	23	14.2
	Neonatology	17	10.5
	Internal Medicine	19	11.7
	Surgery	6	3.7
	Other	7	4.3
	Emergency	19	11.7
	Pharmacy	6	3.7
	Total	162	100.0

A0 = Bachelor degree, A1 = Advanced diploma, A2 = completion of secondary school

### Background information on nurse/midwife managers

With regards to the nurse or midwife managers, 50.3% were A1 nurses while 23% were midwives. Only 20.1% of the nurses and midwives said that their managers benefited from the leadership training while 38.4% said they did not know whether their managers were trained or not. The details of the background information of nurse/midwife managers are presented in Table 2.

**Table 2:** Background information of nurse/midwife managers

Variables	Categories	Frequency	Percent
Training of leadership or management	Yes	32	20.1
	No	66	41.5
	I don’t know	61	38.4
	Total	159	100.0
Highest qualification of nurse manager	Nurse A2	4	2.5
	Nurse A1	81	50.3
	Nurse A0	28	17.4
	Midwife A1	37	23.0
	Midwife A0	3	1.9
	Others	3	1.9
	I don’t know	5	3.1
	Total	161	100.0

### Nurse and midwife managerial leadership styles

The results of this study show that although all four leadership styles of the Path-Goal Leadership tool were exhibited moderately by nurses and midwives in five hospitals of the study, nurse or midwife managers used the directive (autocratic) leadership style (Mean = 18.8, SD = 3.6) more than any other leadership style. This leadership style was followed by the supportive (transformational) leadership style (Mean = 17.3, SD = 4.0) and the participative (democratic) leadership style (Mean = 17.0, SD = 4.1). The leadership style which was least used by nurses and midwives was the achievement-oriented (transactional) (Mean = 15.8, SD = 2.9). The details of the analysis of leadership styles of nurses and midwives managers are presented in Table 3.

**Table 3:** Leadership styles for nurses and midwives’ managers

Leadership styles	Min	Max	Mean	SD
**Directive leadership style**	**12**	**25**	**18.8**	**3.6**
Nurse/mid manager lets team members know what is expected of them	1	5	3.6	1.1
The nurse/mi manager informs team members about what needs to be done and how it needs to be one	1	5	3.7	1.1
The nurse/midwife manager asks team members to follow standard rules and regulations	2	5	4.1	1.0
The nurse/midwife manager explains the level of performance that is expected of team members	1	5	3.8	1.1
The N/M manager gives vague explanations of what is expected of team members on the job (Rev)	1	5	3.6	1.3
**Supportive leadership style**	**6**	**25**	**17.3**	**4.0**
Nurse/mid manager maintains a friendly working relationship with team members	1	5	3.7	1.1
The nurse/midwife manager does little things that make it pleasant to be a member of the team	1	5	3.4	1.1
The nurse midwife manager says things that hut sub-personal feelings (reversed)	1	5	3.5	1.3
The nurse/ midwife manager helps team members overcome problems that stop them from carrying out their tasks	1	5	3.5	1.2
The nurse/midwife manager behaves in a manner that is thoughtful of team members’ personal needs	1	5	3.2	1.2
**Participative leadership style**	**6**	**25**	**17.0**	**4.1**
Nurse/mid manager consults with team members when facing a problem	1	5	3.6	1.2
Nurse/mid manager listens receptively to team members ‘ideas and suggestions	1	5	3.6	1.2
The nurse mid acts without consult team members (Rev)	1	5	2.9	1.2
The nurse/midwife manager asks for suggestions from team members concerning how to carry out assignments	1	5	3.5	1.1
The nurse/midwife manager asks team members for suggestions on what assignments should be made	1	5	3.4	1.3
**Achievement oriented leadership style**	**9**	**22**	**15.8**	**2.9**
The nurse/midwife manager lets team members know that he/she expects them to perform at their highest level	1	5	3.8	1.1
The nurse/midwife manager sets goals for team members’ performance that are quite challenging	1	5	2.8	1.3
The nurse/midwife manager encourages continual improvement in team members ‘performance	1	5	3.5	1.4
The N/MM shows that he/she has doubts about team members’ ability to meet most objectives (Rev)	1	5	3.0	1.3
The nurse/midwife manager consistently sets challenging goals for team members to attain	1	5	2.6	1.3

### Job satisfaction, intention to stay and service provision

The results of this study showed that nurses and midwives had moderate levels of job satisfaction (Mean = 3.68, SD = 0.73). This overlaps with the level of satisfaction of nurses and midwives with their manager’s leadership style (Mean = 3.67, SD = 1). In addition, frontline nurses and midwives were least satisfied with working at their current hospitals until they retire (M = 3.48, SD = 1).

Regarding the intention to stay, the results indicate that the mean intention to stay is 3.42 (SD = 0.79). For service provision, nurses and midwives exhibited slightly higher levels of satisfaction towards the services they were providing (Mean = 3.85 SD = 0.6) and more importantly, they exhibited higher levels of commitment to provide better quality services (Mean = 4.14, SD = 0.7). Details on scores for job satisfaction, intention to stay and service provision are presented in Table 4.

**Table 4:** Job satisfaction, intention to stay and service provision of nurses and midwives

Variables	Min	Max	Mean	SD
**Job satisfaction**	**2**	**5**	**3.68**	**0.730**
I am very satisfied with my job	1	5	3.95	0.911
I feel that my co-workers are satisfied with their jobs	1	5	3.56	0.925
I feel I would be happy to work here until I retire	1	5	3.48	1.065
I feel that the health care facility provides a supportive work environment in which I work	1	5	3.51	1.099
I am very satisfied with my nurse/midwife manager ‘s ability to coordinate activities in the ward	1	5	3.80	0.977
I am very satisfied with my nurse/midwife manager‘s leadership style	1	5	3.67	1.020
I am very satisfied with my relationship with my nurse/midwife manager	1	5	3.81	0.916
**Intention to stay**	**1**	**5**	**3.42**	**0.795**
What are your feelings about your future your hospital (Reversed)	1	5	3.50	1.107
How do you feel about leaving your hospital	1	5	3.27	1.063
If you are free to choose would you prefer to continue working with the hospital (rev)	1	5	3.40	1.133
How important is it to you personally to continue to work with this hospital	1	5	3.52	1.059
**service provision**	**2**	**5**	**3.85**	**0.596**
How can you rate the quality of services provided to the patients in the unit	1	5	3.72	0.766
How can you rate the commitment of the colleagues in provision good health services	1	5	3.78	0.770
How can you rate your commitment to provide better quality services	2	5	4.14	0.704
How can you rate the satisfaction of the patients in your units	2	5	3.73	0.786

### Correlation between leadership styles, job satisfaction, intention to stay and services provision

Table 5 shows the results of Pearson‘s correlation between the study variables. All four managerial leadership styles of Path-Goal leadership theory were positively correlated with nurses’ and midwives’ levels of job satisfaction. However, the achievement-oriented leadership style showed a weak correlation compared to others.

**Table 5:** Correlation between leadership styles and satisfaction, intention to stay and service provision

	Variables	1	2	3	4	5	6
1	Directive style						
2	Supportive style	.726^**^					
3	Participative style	.731^**^	.810^**^				
4	Achievement oriented style	.365^**^	.355^**^	.396^**^			
5	service provision	.389^**^	.200^*^	.244^**^	.253^**^		
6	job satisfaction	.581^**^	.519^**^	.498^**^	.363^**^	.461^**^	
7	intention to stay	.253^**^	.155^*^	.063	.087	.332^**^	.601^**^

* *p* < 0.05; ** *p* < 0.01

For the intention to stay, the results show that there was a significant positive weak relationship between supportive and directive leadership styles and staff intention to stay (r = .15, *p* < 0.001 and r = .25, p < 0.001 respectively). The level of service provisions indicated a positive relationship with all four leadership styles but more with directive than others.

Finally, job satisfaction was strongly correlated with intention to stay (r = .60, *p* < 0.01) and both of them were positively correlated with the level of service provision.

### Relationship between leadership styles, job satisfaction, intention to stay and service provision

A hierarchical multiple linear regression was used to determine how much variation of nurses’/midwives’ job satisfaction, intention to stay, and service provision could be explained by nurse/midwife managerial leadership styles while controlling for demographic characteristics.

Regarding job satisfaction, the first step of the regression analysis indicated that demographic characteristics and background information of the nurse/midwife manager (age of the participants, gender, basic qualification of participants, years of experience with the current manager, experience within the unit, level of the workload and civil status of the nurse/midwife team member) jointly explained 3.3% of the variance in staff level of job satisfaction [*R*^*2*^ = .033]. In the second step, in which nurse and midwife managerial leadership styles (directive, supportive, participative and achievement-oriented) variables were added in the model, it indicated a change of 38% [*R*^*2*^ = .413] (Table 6).

**Table 6:** Relationship between leadership styles and job satisfaction, intention to stay and service provision

Steps	Variables	Job satisfaction				Intention to stay				Service provision			
		Model 1		Model 2		Model 1		Model 2		Model 1		Model 2	
		Beta	*P* value	Beta	*P* value	Beta	*P* value	Beta	*P* value	Beta	*P* value	Beta	*P* value
1	Age	−.059	.670	.009	.935	−.178	.187	−.129	.325	.005	.973	.074	.539
	Gender	.014	.885	.048	.529	.055	.553	.058	.524	.069	.459	.088	.294
	Qualification	.046	.640	−.108	.188	−.078	.422	−.104	.283	.233	.018	.126	.162
	years of experience	.201	.110	.024	.810	.199	.104	.146	.228	−.078	.527	−.213	.057
	experience in the unit	−.072	.470	.071	.387	.107	.270	.143	.139	−.090	.359	.034	.705
	Experience with manager	.074	.450	−.039	.624	−.109	.254	−.147	.117	.006	.952	−.074	.388
	level of workload	.040	.676	−.117	.144	.152	.100	.155	.102	.053	.570	−.022	.805
	civil status	−.061	.576	−.011	.900	.022	.838	.032	.756	.105	.327	.142	.138
2	Directive style			**.400**	**.001**			**.390**	**.005**			**.546**	**.000**
	Supportive style			.178	.180			.142	.362			−.149	.300
	Participative style			.020	.883			**−.384**	**.019**			−.004	.980
	Achievement oriented style			**.201**	**.014**			.081	.395			.136	.124
	**R**^**2**^	.033	.838	**.413**	**.000**	.083	.208	**.183**	**.008**	.074	.289	**.303**	**.000**
	**R**^**2**^**Change**			**.380**				**.100**				**.229**	

Likewise, in the first step, nurses/midwives’ demographic characteristics and background information of the nurse/midwife manager jointly explained 8.3% of the variance in staff level of intention to stay and 7.4% of service provision as perceived by frontline nurses and midwives. In the second step, nurse and midwife managerial leadership styles together significantly predicted the levels of staff intention to stay and service provision, which alone explained 10% [R^2^ = .183] and 22.9% [R^2^ = .303] of the variance in the levels of staff intention to stay and service provision, respectively (Table 6).

## Discussion

This study explored the nurses/midwives managers’ leadership styles and assessed the relationship between leadership styles, job satisfaction, and intention to stay and service provision.

### Nurse/midwife managers’ leadership styles

Consistent leadership is needed to achieve high performance and enhance employees’ capabilities to improve quality of care and clinical outcomes. In nursing and midwifery, leadership is a concept that is important in service delivery because the operation of these services even in a small health facility is extremely complex. Effective managerial leadership styles for nurses and midwives in health facilities are essential to avert errors in clinical practice [26]. The current study revealed that nurse and midwife managers in the five (5) hospitals of the current study used all four managerial leadership styles of the Path-Goal leadership theory depending on the context. Given that the delivery of nursing and midwifery care is a dynamic process, the situational leadership approach has long been identified as useful in these settings to achieve desired clinical outcomes. The situational leadership style is essential in managing specific conditions when need be [27]. This may be the reason that nurses and midwives use all four leadership styles of the Path-Goal leadership theory depending on the context.

Although this study found that nurse and midwife managers use all four leadership styles, the directive leadership style was used most frequently, followed by supportive, participative and then achievement-oriented leadership styles. Under normal circumstances, nurse and midwife managers continue to assist and support their team members. Most of the studies conducted on leadership styles among nurse and midwife managers found that the supportive leadership style was used more commonly and the directive was used less frequently [11, 17, 20, 28]. Many of these studies stressed that nurses and midwives are progressively changing their leadership style from directive to supportive and achievement-oriented leadership styles [11, 20, 28]. In our study setting, in which the directive leadership styles was used most often leaders ask nurses and midwives to follow standards and regulations based on the accreditation process which is an ongoing process in all hospitals in Rwanda.

In order to achieve a higher level of accreditation during the assessment, respect for standards and protocols is essential and therefore, managers must request team members ensure that standards and protocols are respected. This may explain the frequent use of directive leadership style.

### Leadership styles and job satisfaction

This study revealed that job satisfaction of frontline nurses and midwives was moderate. The moderate level of satisfaction reported in this study may be explained by the influence of many motivational factors. These include managerial leadership styles explored in this study and other non-explored factors like remuneration and career development opportunities. In this study, the level of nurses’ and midwives’ satisfaction was shown to be determined by nurses’ and midwives’ managers’ leadership styles. The four leadership styles of Path-Goal theory together explained 38% of the variance in the levels of nurses’ and midwives’ job satisfaction. Additionally, our findings revealed strong positive correlations between job satisfaction and directive leadership style, as well as between job satisfaction and supportive leadership style. This implies that using one or a combination of directive and supportive leadership styles would lead to a corresponding increase in the nursing and midwifery staff level of job satisfaction. Furthermore, a moderate correlation was also found between job satisfaction and participative and achievement-oriented leadership styles.

These findings are consistent with the work of earlier researchers from various contexts which concluded that nursing staff job satisfaction was enhanced by participative and supportive leadership styles [11, 20]. However, Asamani et al. [11] in Ghana found that the achievement-oriented leadership style was positively correlated with nurses’ and midwives’ staff level of satisfaction [11]. We have not found other research that revealed a positive relationship between job satisfaction and the directive leadership style.

The fact that nurses and midwives in hospitals of Rwanda are satisfied with the directive and supportive leadership styles can be explained by a number of factors: (1) directive leadership is the most used leadership style by nurses and midwives managers in hospitals of our study and as a result, it is well known to frontline nurses and midwives. (2) Most hospitals in Rwanda are in the process of accreditation marked by the request to adhere to standards and protocols. Frontline nurses and midwives are asked by their managers to comply with these requests. By doing so, they attain a higher level in accreditation with increased monetary rewards, thereby raising the level of satisfaction. Hospitals where managers use other leadership styles may not achieve a higher level of accreditation, resulting in fewer financial rewards and hence lower financial benefits to staff thus leading to a decrease in staff satisfaction.

Unfortunately, frequent use of a directive leadership style can lead to a decrease in staff satisfaction and an increase in staff turnover, when the enabling factor of using a directive leadership style is no longer there.

### Leadership styles and intention to stay

The literature suggests that there is a direct correlation between a nurse/midwife manager’s leadership style and staff nurse’s/midwife’s intention to stay [9, 11, 17, 20]. This study found that nurses and midwives had moderate intentions to continue to stay at their respective health facilities. The intention to stay for nurses and midwives in this study was higher compared to the one found by Asamani et al. [11] in Ghana as the intention to stay in Ghanaian hospitals was shown to be low (M = 2.65, SD = 0.82). Likewise, 23.6% of nurses and midwives in our study reported that they would prefer not to continue working in their hospitals. This figure is lower than the one found by the study in Ghana as their study reported that 51.7% intended to leave their current hospitals [11]. Likewise, 23.6% intention to leave reported in the current study is relatively small as compared to 67.5% of nurses and midwives who reported to leave in Lebanon [29].

In addition, our results revealed a higher intention to stay (49.4%) compared to the findings of Engeda, Birhanu, and Alene [30] who found 39.8% intent to stay among Ethiopian nurses. However, varying levels of salary were said to be largely responsible for the low intent to stay among the Ethiopian nurses [30]. In the Rwandan context, nurses and midwives benefit from almost the same salary in district hospitals, thus leaving one hospital to work at another would not necessarily lead to improved remuneration and this may explain one of the reasons why intention to leave is slightly lower in our study.

The current study found that nurse and midwife managerial leadership styles together significantly explained 10% of the variance in the levels of staff intention to stay.

Our study revealed significant weak positive relationships between directive and supportive leadership styles with the intention to stay. Our findings are in contrast with those of AbuAlRub and Alghamdi [31] who found no significant relationship between leadership styles and nurses' intention to stay. However, the results are similar to those of Asamani et al. who showed a correlation between intention to stay and leadership styles [11]. The difference between the current study and the earlier study could be attributed to differences in sample characteristics and the cultural context of each country of study. The fact that our study revealed that nurse and midwife managerial leadership styles together significantly explained 10% of the variance in the levels of staff intention to stay is not far from the findings of the study conducted in hospitals of Ghana which reported that nurse managers’ leadership styles jointly explained significant portion (13.3%) of nursing staff intentions to stay in their workplaces [11].

### Leadership styles and staff perceptions on service provision

Service delivery is a process which is comprised of several managerial and employee-specific factors whose inter-relationships and effects upon service quality have been well documented. Employees’ attitudes and behaviours during service delivery play a crucial role in the level of quality of service [32].

Our study revealed that nurses and midwives exhibited slightly higher levels of satisfaction towards the services they were providing and more importantly, they exhibited higher levels of commitment to providing better quality services. A significant moderate relationship between service provision and directive leadership style was found. The nurse and midwife managerial leadership styles together explained 23% of the variance in the levels of services provided. This finding suggests that nurses’ and midwives’ managers should constantly scan their environment to identify their team members’ needs for a particular leadership style to appropriately navigate between the various leadership styles for better service provision. This view is particularly articulated by proponents of the Path-Goal leadership theory [19, 20].

### Implications and future research

Our findings have implications for the nursing and midwifery professions and health system management in general.

First, the study revealed that no one leadership style is ideal for all situations. This implies that to maximize job satisfaction, intention to stay and level of service provision, nurses and midwives managers need to understand their own managerial leadership styles and continuously assess the needs of the frontline staff for whom they are responsible. Nurses and midwives managers do not have sufficient knowledge and skills (only 20% had training on leadership and or management (Table 2) with regards to different leadership styles. There is a need to develop a formal professional continuous development course on leadership and management. All nurses and midwives managers would benefit from this course before or immediately after being appointed as a manager.

Second, the results of this study have significant implications for policymaking in the area of human resources for health specifically for nurses and midwives. The study revealed that nurses and midwives have a moderate level of job satisfaction and a moderate level of intention to stay in their respective hospitals. These findings are in line with the high staff turnover that the health system in the country is facing. Policy intervention is needed to enhance effective leadership in nursing and midwifery in order to address the issue of retention of nurses and midwives in their respective health facilities and to strengthen service provision.

Finally, being cognizant of the role of leadership in health care provision, there is a need to integrate, substantial management and leadership courses into nursing and midwifery education programs curricula.

Researchers in the field of nursing and midwifery management and human resources for health should consider replicating this study in other health facilities including referral hospitals and health centers in Rwanda to provide a holistic view of the subject. Researchers should also consider examining other factors affecting staff job satisfaction, intention to stay and service provision in the context of Rwanda considering that the current study only examined the influence of leadership styles on these three concepts.

### Strength and limitation of the study

This study was conducted in five hospitals located in one province of Rwanda out of five provinces to give an overview of current leadership styles used by nurses and midwives managers to determine how they influence job satisfaction, intention to stay and service provision. Given the differences between the hospitals included in this study (rural vs urban, and public vs faith-based), the findings can be generalized to other hospitals in Rwanda, particularly provincial and district hospitals.

Based on the cross-sectional design used in this study, there may be a social desirability bias. However, given that the questionnaires were self-administered, anonymous, and nurses/midwives managers did not have contact with the completed questionnaires, it is probable that the respondents have provided honest and complete information. To complement the findings of this study, a qualitative study is needed in order to provide a deeper understanding of why the nurses and midwives report moderate job satisfaction and intentions to stay.

## Conclusion

This study aimed to explore the leadership styles adopted by nurses’ and midwives’ managers and their influences on nurses’ and midwives’ job satisfaction, intention to stay as well as the provision of services in five hospitals of Rwanda. Knowledge in this area is currently limited within the country. Although moderate scores were recorded for all four leadership styles of the Path-Goal Leadership tool, nurse and midwife managers used the directive leadership style more than any other leadership style. This was followed by supportive and participative styles, while the least used was achievement-oriented. Nurses and midwives in this study exhibited moderate levels of job satisfaction and moderate intentions to continue to stay in their current hospitals. However, they exhibited slightly higher levels of satisfaction towards services they are providing. Furthermore, leadership styles were associated with job satisfaction, intention to stay and service provision. The findings of this study, suggest that nurses and midwife leadership should be enhanced at all levels to improve nursing and midwifery staff job satisfaction, retention and level of perceived service provision.

## Data Availability

The datasets used in this study may be available from the corresponding author upon a reasonable request.

## Abbreviations

CMHS-IRB: College of Medicine and Health Sciences Institutional Review Board
DN: Director of Nursing
PI: Principal Investigator
